# Association Between Dynamic Changes in the Sling-Pubis Gap and Urinary Symptoms After Tension-Free Vaginal Tape Surgery: Protocol for a Prospective Observational Pilot Study

**DOI:** 10.2196/95847

**Published:** 2026-10-20

**Authors:** Yaman Degirmenci, Ceren Efe Sayın, Annette Hasenburg, Gilbert Georg Klamminger

**Affiliations:** 1 Department of Gynecology and Obstetrics, University Medical Center of the Johannes Gutenberg University, Langenbeck. Str. 1, Mainz, 55131, Germany, +49 6131 17 ext. 1; 2 Department of Biostatistics and Medical Informatics, Faculty of Medicine, Kahramanmaraş Sütçü İmam University, Kahramanmaraş, Türkiye

**Keywords:** stress urinary incontinence, midurethral sling, transperineal ultrasound, sling-pubis gap, International Prostate Symptom Score, IPSS, voiding dysfunction, pelvic floor, prospective study

## Abstract

**Background:**

Stress urinary incontinence (SUI) is one of the most common pelvic floor disorders in women and is frequently treated surgically using midurethral sling procedures. Despite the high success rates of these procedures, postoperative voiding dysfunction remains a clinically relevant complication that may negatively affect patient satisfaction and quality of life. The underlying mechanisms are multifactorial and not completely understood. In particular, the interaction among sling position, urethral mobility, and pelvic floor dynamics during increases in intra-abdominal pressure may influence postoperative urinary function. Dynamic transperineal ultrasound has emerged as a valuable imaging modality for evaluating sling position and urethral mobility; however, the relationship between dynamic sonographic sling parameters and postoperative urinary symptoms has not yet been sufficiently clarified.

**Objective:**

This study aims to investigate the association between dynamic sonographic sling parameters and postoperative changes in patient-reported lower urinary tract symptoms following retropubic tension-free vaginal tape surgery.

**Methods:**

This prospective, noninterventional observational pilot study will be conducted at the Department of Obstetrics and Gynecology, University Medical Center Mainz, Germany. Female patients scheduled for retropubic tension-free vaginal tape surgery for SUI will be recruited consecutively. Preoperative assessment will include clinical examination, completion of the International Prostate Symptom Score (IPSS) questionnaire, and standardized transperineal ultrasound imaging at rest and during the Valsalva maneuver. Postoperative follow-up will be performed 3 months after surgery and will include repeat ultrasound examination and IPSS assessment. Sonographic parameters will include urethral mobility, sling-pubis gap, tape-urethra distance, and sling morphology. The primary outcome will be the correlation between changes in the sling-pubis gap during the Valsalva maneuver and changes in IPSS scores. Secondary outcomes will include associations among clinical variables, sonographic findings, and postoperative voiding symptoms.

**Results:**

Ethics approval for this study has been obtained from the Ethics Committee of the State Medical Association of Rhineland-Palatinate (reference number 2025-18448). At the time of manuscript submission, participant recruitment had not yet begun and was scheduled to begin in August 2026. The target sample size for this pilot study is 55 participants. Data collection is expected to take approximately 24 months, and the final study results are anticipated to be available in 2028.

**Conclusions:**

This study will investigate dynamic sonographic parameters associated with postoperative urinary symptoms following midurethral sling surgery. A better understanding of sling-urethra interactions during increases in intra-abdominal pressure may improve the prediction of postoperative voiding dysfunction and contribute to more individualized surgical planning and patient counseling in women undergoing treatment for SUI.

## Introduction

Stress urinary incontinence (SUI), defined as the involuntary leakage of urine during physical exertion, affects nearly one-third of women and is strongly associated with a substantial reduction in quality of life, often leading to social isolation, emotional distress, and impaired sexual well-being [1-3]. The etiology of SUI is multifactorial; however, prevailing pathophysiological theories primarily attribute its development to urethral hypermobility resulting from loss of urethral suspension, suburethral support, and urethral resistance [4,5]. Accordingly, treatment strategies primarily aim to restore urethral support and suspension.

Since their introduction in the 1990s based on the integral theory, which emphasizes the importance of adequate urethral stability for maintaining continence, midurethral slings (MUSs) have become the standard first-line surgical treatment for SUI [6-9]. The continence mechanism of MUSs relies on dynamic urethral support: the sling functionally replaces the pubourethral ligaments and compresses the urethra against the pubic symphysis during increases in intra-abdominal pressure, resulting in urethral kinking and restoration of continence [10-12]. Objective success rates of sling procedures are compelling, with long-term cure rates of approximately 90% [13,14]. The original placement technique recommends positioning the tape beneath the middle third of the urethra, corresponding to the high-pressure zone and the urethral knee, where the pubourethral ligaments attach [7,15]. The goal of MUS placement is a tension-free insertion of the sling in a suburethral position, with intraoperative adjustment possible using maneuvers such as the cough test [16,17].

Despite its high success rates, MUS surgery may be associated with complications related to the therapeutic urethral obstruction induced by the sling, including postoperative voiding dysfunction—such as hesitancy, weak urinary stream, intermittent voiding, or straining to void—as well as overactive bladder symptoms, including urgency, frequency, and urgency urinary incontinence [18-22]. The reported incidence of voiding dysfunction varies widely, ranging from single-digit percentages to a prevalence as high as 60% depending on how voiding dysfunction is defined and the diagnostic criteria applied [18,23]. Beyond its negative impact on quality of life and the frustration experienced by patients who expect a curative outcome, postoperative voiding dysfunction may also lead to long-term complications affecting both the lower and upper urinary tracts.

Several preoperative predictors of postoperative voiding dysfunction have been proposed, including prior incontinence surgery, concomitant prolapse surgery, and urodynamic parameters such as maximum flow rate (Q_max_). However, the available evidence regarding these predictors remains limited and inconsistent [22,24]. Consequently, there is growing interest in imaging techniques that allow for the dynamic assessment of sling position and function.

Owing to its excellent interobserver agreement, pelvic floor ultrasound has gained increasing importance in urogynecological practice and has become an established tool for evaluating sling position, urethral mobility, and mesh-related complications [25-27]. Sonography enables a detailed assessment of the MUS position and dynamic behavior after its placement. Several sonographic parameters have been proposed as potential predictors of surgical outcomes. The sling-pubis gap (SPG), defined as the distance between the sling and the pubic symphysis, was first described more than a decade ago and has been suggested to correlate with MUS outcomes. Studies suggest that a relatively small SPG may be associated with successful sling function, whereas an excessively small SPG may indicate excessive urethral compression and has been linked to postoperative voiding dysfunction [28]. Similarly, postoperative sonographic parameters, including the sling-urethra distance and sling configuration, may reflect postoperative sling tension and have been suggested to be associated with postoperative urinary symptoms and urodynamic parameters [15,29,30]. Although the position of the tape relative to the urethra may also affect surgical outcomes, this relationship has not been consistently confirmed across studies, particularly with regard to urethral length or specific urethral segments [18,26,31].

Nevertheless, evidence regarding these sonographic parameters remains conflicting. The Valsalva maneuver, a forced expiratory effort against a closed airway that transiently increases intra-abdominal pressure, is commonly used during pelvic floor ultrasound to assess dynamic changes in pelvic floor anatomy and urethral mobility. The reported distributions of SPG values during the Valsalva maneuver overlap considerably between patients with and without incontinence, and several studies have failed to demonstrate a clear association between SPG and surgical outcomes [32-35]. Consequently, universally accepted cutoff values have not yet been established [2]. The overlapping distributions highlight the limited discriminatory value of these parameters for individual patients.

While the exact tape location relative to the middle third of the urethra remains debated, sling tension is widely accepted as a key factor influencing both surgical success and complication rates [36]. However, the considerable anatomical and functional variability among patients suggests that a “one-size-fits-all” approach to sling tensioning may not be appropriate.

Functional characteristics of the lower urinary tract may also influence postoperative outcomes. Approximately 30% of women without prior incontinence surgery may demonstrate abnormal uroflowmetry findings despite the absence of corresponding symptoms [23]. Furthermore, nearly half of women void using Valsalva-assisted abdominal straining, which has been associated with an increased risk of postoperative voiding dysfunction following MUS surgery [37,38]. Women with detrusor underactivity frequently rely on abdominal straining during micturition, and parameters reflecting detrusor underactivity, such as a low maximum detrusor pressure during voiding (P_detmax_) × average urinary flow rate during voiding (Q_ave_) value, have been proposed to predict increased postoperative postvoid residual (PVR) volumes and voiding difficulties, although the available evidence remains inconsistent [19,39]. Taken together, these findings highlight the importance of individualized dynamic evaluation and comprehensive preoperative functional assessment when predicting postoperative outcomes.

Patient-reported outcome measures play an important role in evaluating postoperative urinary symptoms. Among the available instruments, the International Prostate Symptom Score (IPSS) has been widely used to quantify the severity of lower urinary tract symptoms. Recently, the IPSS has been shown to demonstrate excellent internal consistency and good construct validity in assessing female lower urinary tract symptoms [40]. Although originally developed for benign prostatic hyperplasia, the IPSS has proven to be a useful tool for evaluating lower urinary tract symptom severity in women [41]. Linking dynamic sonographic sling parameters with patient-reported symptom changes may provide important insights into the functional mechanisms underlying postoperative voiding dysfunction.

Epidemiological data indicate that more than 1 in 10 women will undergo surgical treatment for SUI during their lifetime [42]. Despite increasing interest in the sonographic assessment of sling position, the relationship between pre- and postoperative sonographic parameters and patient-reported voiding symptoms remains poorly understood, and prospective studies evaluating this relationship are scarce. A better understanding of these associations may improve perioperative risk stratification and help optimize sling placement strategies, potentially reducing postoperative voiding dysfunction. Therefore, this prospective study aims to investigate whether preoperative urethral mobility and postoperative sonographic sling parameters—including the SPG, sling-urethra distance, urethrovesical angle, and sling configuration—are associated with postoperative changes in patient-reported voiding symptoms measured using the IPSS.

## Methods

### Study Design and Setting

This prospective, noninterventional observational pilot study aims to investigate the relationship between dynamic sonographic sling parameters and postoperative changes in lower urinary tract symptoms following retropubic tension-free vaginal tape (TVT) surgery. The study will be conducted at the Department of Obstetrics and Gynecology, University Medical Center Mainz, Germany. The study protocol was developed in accordance with the SPIRIT (Standard Protocol Items: Recommendations for Interventional Trials) guidelines where applicable and adapted to the observational design [43,44]. Each participant will be followed for 3 months after surgery, and the total study duration is expected to be approximately 24 months. The study has been registered in the German Clinical Trials Register under the identifier DRKS00038954. Participants will be recruited from the urogynecological outpatient clinic of University Medical Center Mainz, a tertiary referral center. All diagnostic procedures and follow-up examinations will be performed at the same institution, ensuring standardized assessment conditions and imaging protocols.

### Inclusion Criteria

Participants must meet the following criteria: female patients aged 18 to 80 years, planned retropubic TVT surgery for SUI, preoperative PVR volume of 100 mL or less, ability to provide written informed consent, and participation in routine postoperative follow-up.

### Exclusion Criteria

Participants will be excluded if any of the following criteria are present: postoperative PVR volume above 100 mL, requirement for sling loosening or reoperation, neurological disorders affecting bladder function, pregnancy, lack of informed consent, previous MUS surgery or major urethral surgery, and ongoing unstable therapy with anticholinergic or β_3_-adrenoceptor agonist medication. Only German-speaking participants will be included to ensure reliable completion of the questionnaire-based assessments.

Patients with a postoperative PVR volume greater than 100 mL will be excluded because this value exceeds the clinically accepted range after TVT surgery and prompts evaluation for clinically relevant outlet obstruction and possible sling loosening or revision according to routine clinical management. Such patients represent a distinct postoperative complication group rather than the functional spectrum of uncomplicated sling-urethra interactions targeted in this study. Their inclusion could confound the association between dynamic sling position and symptom change because subsequent clinical intervention may alter both sling anatomy and postoperative symptoms.

### Recruitment

Participants will be recruited consecutively from patients presenting to the urogynecological outpatient clinic who are scheduled for retropubic TVT surgery. Eligible patients will receive detailed information about the study during the preoperative consultation, and written informed consent will be obtained before enrollment.

### Study Procedures

Table 1 summarizes all sonographic parameters and their definitions.

**Table 1.** Sonographic parameters, definitions, timing of measurement, and analytical purpose.

Parameter	Definition	Time point	Purpose
SPG^a^	Distance between the sling and the tip of the pubic symphysis	At rest and during the Valsalva maneuver	Primary variable
Tape-urethra distance	Shortest distance between the tape and the LSM^b^ complex	At rest only	Secondary parameter
Urethra-pubis distance	Distance from the LSM complex to the pubis	Preoperatively	Anatomical reference
ΔSPG	Difference between SPG at rest and during the Valsalva maneuver	Calculated	Dynamic mobility

a SPG: sling-pubis gap.

b LSM: longitudinal smooth muscle.

#### Baseline Assessment

Prior to surgery, all participants will undergo a routine clinical examination and a standardized transperineal ultrasound examination and complete the IPSS questionnaire. Participants will perform Valsalva maneuvers lasting at least 6 seconds, repeated 3 times during the ultrasound examination to ensure reproducibility.

#### Ultrasound Measurements, Sling Position, and Morphology Assessment

Examinations will be performed using a transperineal convex transducer with the patient in the dorsal lithotomy position after spontaneous bladder emptying by an experienced urogynecologist.

Midsagittal 2D transperineal ultrasound images of the pelvic floor will be obtained to evaluate urethral mobility, sling position, sling morphology, and dynamic changes in the spatial relationship among the urethra, the sling, and the tip of the pubic symphysis. Preoperatively, ultrasound images will be obtained at rest and during a standardized Valsalva maneuver. Before image acquisition, participants will receive standardized verbal instructions to perform a maximal Valsalva maneuver lasting at least 6 seconds. Three Valsalva maneuvers will be performed, and the examination demonstrating adequate pelvic floor descent will be used for analysis. Participants unable to perform an adequate Valsalva maneuver despite repeated instruction will be excluded from the study. All ultrasound images will be stored and analyzed offline using predefined anatomical landmarks. SPG measurements will be performed independently by 2 investigators blinded to the participants’ clinical characteristics and postoperative IPSS results. If the measurements differ by more than 2 mm, a third measurement will be performed, and the mean value will be used for analysis. Interobserver agreement will be assessed using the intraclass correlation coefficient.

During preoperative image analysis, the shortest distance between the hyperechogenic dorsal margin of the urethra—corresponding to the longitudinal smooth muscle (LSM) complex of the urethra—and the tip of the pubic symphysis will be measured. This measurement represents the preoperative urethra-pubis distance and will serve as the anatomical reference value for further analysis. Because the exact position of the sling cannot be predicted preoperatively, the urethra will be divided into 3 anatomical segments (proximal, middle, and distal thirds) to standardize measurement levels.

After completion of the postoperative examination, the final sling position relative to the urethra will first be identified using postoperative ultrasound images. Subsequently, the corresponding anatomical level on the preoperative ultrasound images will be determined, and measurements will be obtained at the same urethral segment to ensure anatomical comparability between preoperative and postoperative measurements. During postoperative assessment, the SPG will be measured on midsagittal ultrasound images as the distance from the center of the MUS to the tip of the pubic symphysis, as described by Taithongchai et al [26]. This measurement will be obtained during the Valsalva maneuver (Figure 1).

**Figure 1.**
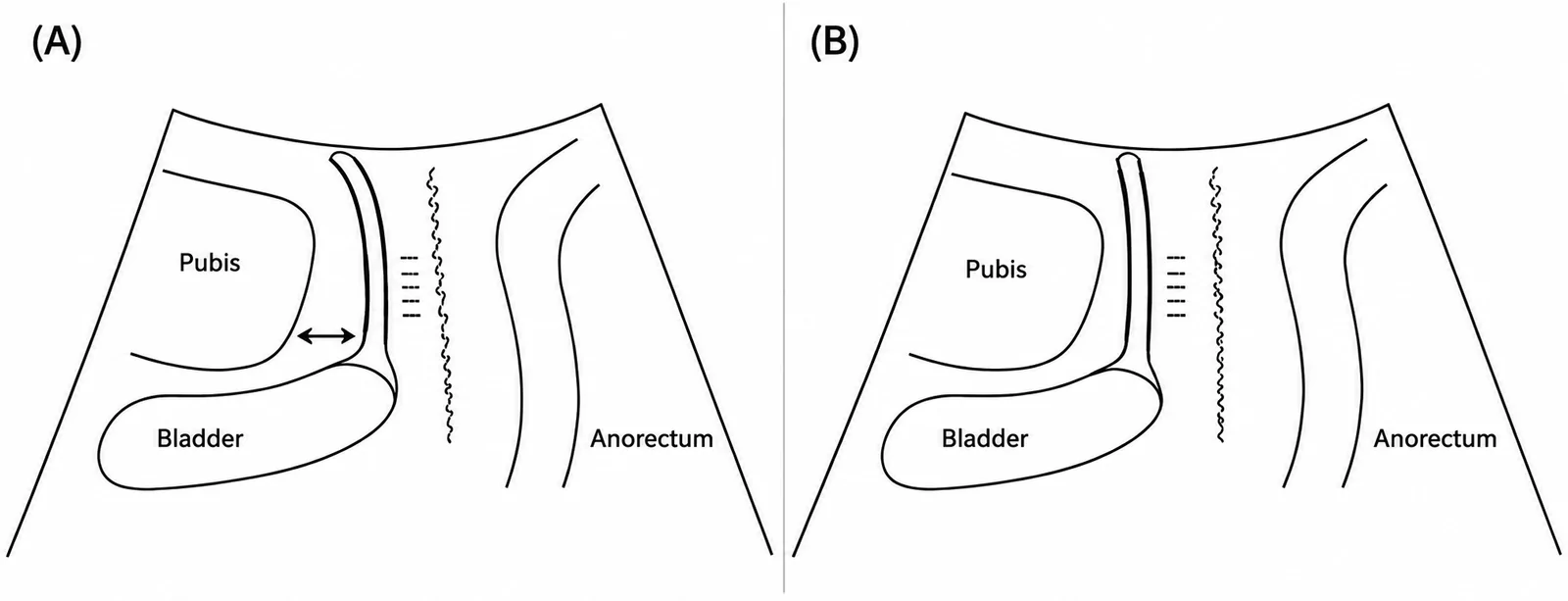
Schematic illustration of the measurement principle used for assessment of the urethra-pubis distance and the sling-pubis gap (SPG). The diagrams illustrate the anatomical reference points used for perineal ultrasound measurements at rest (B) and during the Valsalva maneuver (A). Preoperatively, because the final sling position is unknown, the reference point corresponds to the hyperechogenic dorsal wall of the urethra (longitudinal smooth muscle complex) at the urethral level where the sling is subsequently located on postoperative ultrasound. Postoperatively, the same anatomical level is identified, and the center of the midurethral sling serves as the reference point. In both conditions, the shortest linear distance to the tip of the pubic symphysis is measured. This approach enables direct anatomical comparison between the preoperative urethra-pubis distance and the postoperative SPG both at rest and during the Valsalva maneuver.

The relative change between the preoperative urethra-pubis distance and the postoperative SPG measured during the Valsalva maneuver will be calculated according to the following formula:

SPG change (%) = [(preoperative urethra-pubic symphysis gap – postoperative SPG)/preoperative urethra-pubic symphysis gap] × 100

This parameter reflects the dynamic interaction between the sling and the urethra during increased intra-abdominal pressure. All ultrasound images will be analyzed offline by investigators blinded to clinical outcomes and IPSS results.

#### Calculation of Dynamic Urethral Displacement

To facilitate the interpretation of urethral mobility, changes in urethral displacement during the Valsalva maneuver before and after surgery will also be evaluated. To facilitate interpretation of urethral mobility, displacement during the Valsalva maneuver will be calculated as the difference between measurements obtained at rest and during the Valsalva maneuver. This calculation will be performed both preoperatively (urethra-pubis distance) and postoperatively (SPG). Relative displacement will also be expressed as the percentage change from the resting measurement, allowing for the comparison of dynamic mobility before and after surgery. A reduction in displacement after surgery will be interpreted as reduced urethral mobility due to stabilization by the MUS.

#### Sling Morphology Assessment

Sling morphology will be categorized according to previously described ultrasound classifications [26]: flat configuration both at rest and during the Valsalva maneuver (type 1), flat configuration at rest and C- or U-shaped configuration during the Valsalva maneuver (type 2), and C- or U-shaped configuration both at rest and during the Valsalva maneuver (type 3).

#### Tape-Urethra Distance

The tape-urethra distance will be measured at rest according to the method described by Kociszewski et al [15], defined as the shortest distance between the tape and the urethra’s LSM complex. Because the tape-urethra distance is conventionally measured at rest and no standardized method has been established for its assessment during the Valsalva maneuver, the preoperative urethra-pubis distance measured at the level of the LSM complex will be used as the anatomical reference for the postoperative SPG, allowing for the estimation of the relative displacement of the sling-urethra complex during the Valsalva maneuver. Therefore, only the resting tape-urethra distance will be included in the analysis.

### Surgical Procedure

All patients will undergo a retropubic TVT procedure performed according to the standardized surgical technique described by Ulmsten et al [16]. The procedure will be carried out by urogynecological surgeons at the study center. After bladder emptying via a transurethral catheter, a midline vaginal incision will be created in the suburethral vaginal wall, followed by blunt dissection to develop the paraurethral tunnels. The TVT needle will then be advanced through the retropubic space until it exits through the suprapubic skin incision. Intraoperative cystoscopy will be performed to exclude bladder injury. The sling will be positioned tension-free beneath the midurethra, and the vaginal incision will be closed according to standard surgical practice.

### Patient-Reported Outcome Measures

#### Overview

Lower urinary tract symptoms will be assessed using the German version of the IPSS questionnaire [45]. The questionnaire evaluates voiding symptoms, storage symptoms, and quality of life related to urinary symptoms. IPSS scores will be recorded preoperatively and at the 3-month postoperative follow-up. Changes in symptom severity will be expressed as the difference between postoperative and preoperative IPSS scores (ΔIPSS).

#### Primary Outcome

This is the correlation between the percentage change in the SPG during the Valsalva maneuver and the change in the IPSS.

The primary outcome was defined as the change in the IPSS because the objective of the study is to investigate the relationship between dynamic anatomical changes in sling position and changes in patient-reported urinary symptoms. Using the ΔIPSS as a continuous outcome avoids the loss of information associated with dichotomizing postoperative voiding dysfunction into present or absent categories and is therefore better suited for correlation analyses in this exploratory pilot study. Furthermore, the ΔIPSS reflects the magnitude of symptom change and, thus, more comprehensively captures the clinical impact of postoperative voiding dysfunction.

#### Secondary Outcomes

These include the association between ΔIPSS and clinical parameters (age, BMI, baseline IPSS, and sling position) as well as sonographic parameters, including the urethrovesical angle and the postoperative tape-urethra distance; interobserver reliability of ultrasound measurements; and exploratory identification of a ΔIPSS of 4 or higher as an indicator of clinically relevant voiding dysfunction.

#### Schedule of Study Assessments

The schedule of study assessments at baseline and at the 3-month postoperative follow-up is summarized in Table 2.

**Table 2.** Study schedule.

Assessment	Baseline	3 months
Clinical examination	✓	✓
IPSS^a^	✓	✓
Transperineal ultrasound	✓	✓
Sling morphology		✓
SPG^b^		✓

a IPSS: International Prostate Symptom Score.

b SPG: sling-pubis gap.

### Sample Size Calculation

No previous study has directly evaluated the association between dynamic changes in the SPG and postoperative changes in the IPSS. Therefore, the anticipated effect size could not be derived from an identical prior study. For this exploratory pilot study, a correlation coefficient of *r*=0.40 was prespecified as a clinically meaningful moderate association. Using a 2-sided significance level of α=.05 and 80% power, 47 evaluable participants are required to detect a correlation of this magnitude. Assuming a 15% attrition or nonevaluable data rate, the planned sample size was increased to 55 participants.

### Statistical Analysis

Statistical analyses will be performed using SPSS (IBM Corp) or R (R Foundation for Statistical Computing). The normality of continuous variables will be assessed using the Shapiro-Wilk test together with visual inspection of histograms. Descriptive statistics will be presented as means and SDs for normally distributed variables, medians and IQRs for nonnormally distributed variables, and frequencies with percentages for categorical variables. Associations between the percentage change in the SPG during the Valsalva maneuver and ΔIPSS will be evaluated using the Pearson or Spearman correlation coefficients depending on data distribution. Associations between ΔIPSS and clinical and sonographic parameters, including the urethrovesical angle and postoperative tape-urethra distance, will first be explored using univariable analyses and subsequently assessed in multivariable linear regression models. Interobserver reliability of ultrasound measurements will be evaluated using the intraclass correlation coefficient. As part of an exploratory analysis, clinically relevant voiding dysfunction will be defined as a ΔIPSS of 4 or higher, and factors associated with this outcome will also be explored using logistic regression. Statistical significance will be defined as a *P* value below .05.

### Missing Data

Missing data will be minimized through standardized follow-up procedures and immediate verification of study forms at each visit. The primary analysis will be based on complete cases. Participants with missing primary outcome data (ΔIPSS) or nonevaluable ultrasound measurements required for SPG calculation will be excluded from the primary correlation analysis. The number and reasons for missing or nonevaluable data will be reported. As this is an exploratory pilot study, no imputation of missing data is planned.

### Ethical Considerations

All data will be pseudonymized and stored on a secure institutional server. The key linking patient identifiers and study codes will be stored separately. Data will be retained for 10 years after study completion. Participants may withdraw from the study at any time without providing a reason.

The study will be conducted in accordance with the Declaration of Helsinki. Ethics approval was granted by the Ethics Committee of the State Medical Association of Rhineland-Palatinate (reference number 2025-18448). The study has been registered in the German Clinical Trials Register (DRKS00038954). Written informed consent will be obtained from all participants before inclusion in the study. No financial compensation will be provided for participation.

## Results

At the time of manuscript submission, participant recruitment had not yet begun and was scheduled to begin in August 2026 at the Department of Obstetrics and Gynecology, University Medical Center Mainz. The planned sample size for this pilot study is 55 participants. Data collection is expected to take approximately 24 months, including patient recruitment, surgical procedures, and the scheduled 3-month postoperative follow-up assessments. Data analysis will commence after completion of follow-up and verification of the study database. The final study results are expected to be available in 2028. The study findings will be disseminated through peer-reviewed publications and presentations at scientific conferences. A study flow diagram illustrating participant recruitment, eligibility assessment, and follow-up procedures is shown in Figure 2.

**Figure 2.**
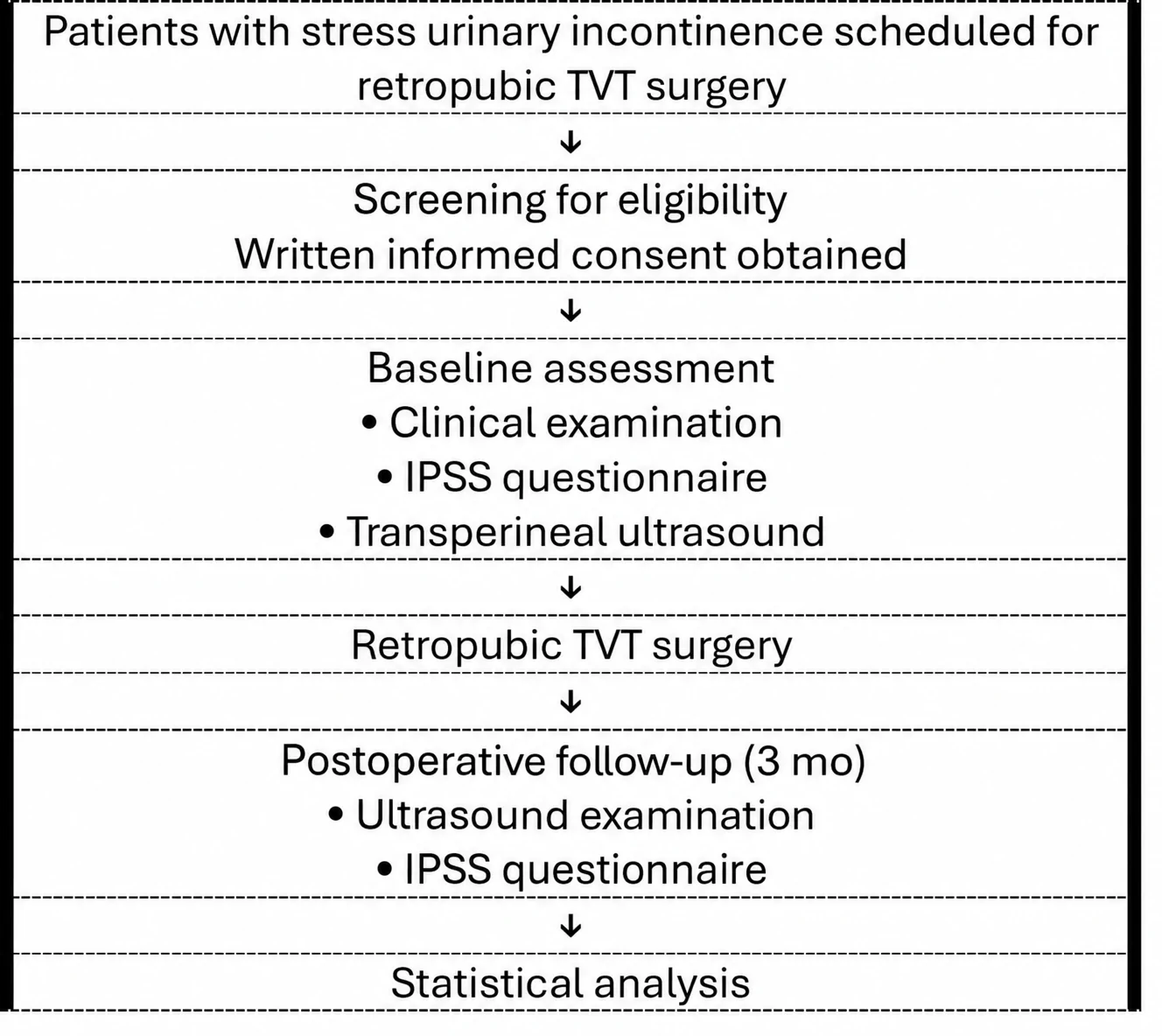
Study flow diagram. IPSS: International Prostate Symptom Score; TVT: tension-free vaginal tape.

## Discussion

### Principal Findings

SUI is one of the most common pelvic floor disorders in women and frequently requires surgical treatment using MUS procedures [3,42]. These procedures have become the standard surgical treatment due to their high success rates and relatively low invasiveness. However, despite their overall safety and effectiveness, postoperative voiding dysfunction remains a clinically relevant complication [37]. Even though the procedure is typically performed in a minimally invasive and often outpatient setting, postoperative voiding difficulties may negatively affect patient satisfaction and quality of life.

Many surgeons have likely encountered patients in whom the sling appeared to be correctly positioned and tension-free intraoperatively, yet postoperative voiding dysfunction still developed. The mechanisms underlying postoperative voiding dysfunction are multifactorial and not yet fully understood. In particular, the interaction among sling position, urethral mobility, and dynamic pelvic floor mechanics during increases in intra-abdominal pressure may play an important role [5,11,12]. A better understanding of these anatomical and functional relationships could contribute to improved prediction of postoperative symptoms.

Identifying sonographic parameters associated with postoperative voiding dysfunction may therefore help optimize surgical outcomes and support more individualized preoperative counseling of patients [26,29,33]. The present study aims to explore the relationship between dynamic sonographic sling parameters and postoperative changes in patient-reported urinary symptoms. By combining objective imaging findings with symptom-based outcome measures, this study may provide new insights into the functional mechanisms underlying voiding symptoms after MUS surgery. The findings may contribute to improved perioperative risk stratification and potentially guide more individualized sling placement strategies.

### Strengths and Limitations of This Study

A major strength of this study is its prospective design and the standardized assessment of both imaging parameters and patient-reported outcomes. The use of clearly defined anatomical landmarks such as the LSM complex of the urethra and the tip of the pubic symphysis allows for reproducible sonographic measurements. In addition, independent ultrasound assessments and interobserver reliability analysis will enhance measurement robustness.

Another methodological strength is the systematic classification of sling morphology based on previously described ultrasound patterns. The classification into flat, C-shaped, or U-shaped configurations at rest and during the Valsalva maneuver allows for the investigation of potential associations between sling configuration and postoperative voiding symptoms.

However, limitations should be acknowledged. The study will be conducted at a single tertiary referral center and include a relatively small sample size, reflecting its pilot nature. Although the intended MUS position can be achieved in most patients, variations in sling placement within the proximal or distal thirds of the urethra may lead to different functional outcomes and introduce heterogeneity into the analysis.

In addition, the absence of preoperative and postoperative urodynamic testing may limit the ability to identify underlying functional abnormalities that could contribute to voiding dysfunction or influence postoperative outcomes. Although the IPSS questionnaire has been validated, it represents a patient-reported outcome measure and, therefore, inherently contains subjective components [41,45]. Furthermore, the 3-month follow-up period focuses primarily on early postoperative outcomes and may not capture longer-term functional changes after sling surgery. Although the Valsalva maneuver will be standardized during ultrasound examinations, some patients may have difficulty performing it consistently, which could influence measurements. Despite duplicate measurements and interobserver reliability analysis, patient cooperation during Valsalva maneuvers and image interpretation may still introduce variability in measurements.

Finally, the study population is limited to German-speaking participants because the validated German version of the IPSS questionnaire will be used to assess urinary symptoms. This restriction may limit the generalizability of the findings to broader international populations.

### Future Directions

This study proposes a dynamic sonographic assessment that links preoperative urethral mobility to postoperative sling behavior. If significant associations between sonographic sling parameters and postoperative symptoms are identified, future studies with larger multicenter cohorts and longer follow-up periods may further validate these findings. Ultimately, an improved understanding of sling dynamics may contribute to more personalized surgical strategies in the treatment of SUI.

## Supplementary material

10.2196/95847 Checklist 1 SPIRIT checklist.

## Abbreviations

IPSS: International Prostate Symptom Score
LSM: longitudinal smooth muscle
MUS: midurethral sling
PVR: postvoid residual
SPG: sling-pubis gap
SPIRIT: Standard Protocol Items: Recommendations for Interventional Trials
SUI: stress urinary incontinence
TVT: tension-free vaginal tape
