# Supplementary material for: Association Between Dynamic Changes in the Sling-Pubis Gap and Urinary Symptoms After Tension-Free Vaginal Tape Surgery: Protocol for a Prospective Observational Pilot Study
Source: JMIR Res Protoc. 2026 Oct 20;15:e95847. doi: 10.2196/95847 (PMC13682679; doi:10.2196/95847)
Supplement: Supplementary file 1 [file resprot-v15-e95847-s001.pdf]

Spirit-Checklist-Spindle-Study

SPIRIT Item	Description	Manuscript Section
1	Descriptive title	Title page
2a	Trial registration	Methods – Study Design and Setting
2b	WHO Trial Registration Data Set	Methods – Study Design and Setting
3	Protocol version	Title page
4	Funding	Funding
5a	Roles and responsibilities	Author Contributions
5b	Name of sponsor	Not applicable (no external sponsor)
5c	Role of sponsor	Not applicable (no external sponsor)
6	Background and rationale	Introduction
7	Objectives	Introduction / Outcome Measures
8	Trial design	Study Design and Setting
9	Study setting	Study Design and Setting
10	Eligibility criteria	Inclusion and Exclusion Criteria
11a	Interventions	Surgical Procedure
11b	Criteria for discontinuing interventions	Exclusion Criteria
11c	Strategies to improve adherence	Not applicable (observational study)
11d	Concomitant care	Not applicable (routine clinical care)
12	Outcomes	Outcome Measures
13	Participant timeline	Table 2 – Study Schedule
14	Sample size	Sample Size Calculation
15	Recruitment	Recruitment
16a	Sequence generation	Not applicable
16b	Allocation concealment	Not applicable
16c	Implementation	Not applicable
17a	Blinding	Ultrasound Measurements
17b	Unblinding	Not applicable
18a	Data collection methods	Baseline Assessment / Ultrasound / IPSS

SPIRIT Item	Description	Manuscript Section
18b	Participant retention	Missing Data
19	Data management	Data Management
20a	Statistical methods	Statistical Analysis
20b	Additional analyses	Statistical Analysis
20c	Missing data analysis	Missing Data
21	Data monitoring	Not applicable
22	Harms	Ethical Considerations
23	Auditing	Not applicable
24	Research ethics approval	Ethical Considerations
25	Protocol amendments	Ethical Considerations
26a	Consent	Recruitment / Ethical Considerations
26b	Additional consent	Not applicable
27	Confidentiality	Data Management
28	Declaration of interests	Conflicts of Interest
29	Access to data	Data Availability
30	Ancillary and post-trial care	Not applicable
31a	Dissemination policy	Results / Data Availability
31b	Authorship	Author Contributions
31c	Public access to protocol/data	Data Availability
32	Informed consent materials	Not applicable
33	Biological specimens	Not applicable
